# ERK phosphorylation of MED14 in promoter complexes during mitogen-induced gene activation by Elk-1

**DOI:** 10.1093/nar/gkt837

**Published:** 2013-09-17

**Authors:** Matthew D. Galbraith, Janice Saxton, Li Li, Samuel J. Shelton, Hongmei Zhang, Joaquin M. Espinosa, Peter E. Shaw

**Affiliations:** ^1^School of Biomedical Sciences, University of Nottingham, Queen’s Medical Centre, Nottingham, NG7 2UH, UK, ^2^Department of Molecular, Cellular and Developmental Biology, Howard Hughes Medical Institute, University of Colorado at Boulder, Boulder, CO 80309, USA, ^3^Department of Neurology, Center of Regeneration Medicine and Stem Cell Research, University of California, San Francisco, CA 90089, USA and ^4^Department of Pharmacology, Ningxia Medical University, Yinchuan 750004, China

## Abstract

The ETS domain transcription factor Elk-1 stimulates expression of immediate early genes (IEGs) in response to mitogens. These events require phosphorylation of Elk-1 by extracellular signal-regulated kinase (ERK) and phosphorylation-dependent interaction of Elk-1 with co-activators, including histone acetyltransferases and the Mediator complex. Elk-1 also recruits ERK to the promoters of its target genes, suggesting that ERK phosphorylates additional substrates in transcription complexes at mitogen-responsive promoters. Here we report that MED14, a core subunit of the Mediator, is a *bona fide* ERK substrate and identify serine 986 (S986) within a serine-proline rich region of MED14 as the major ERK phosphorylation site. Mitogens induced phosphorylation of MED14 on S986 at IEG promoters; RNAi knockdown of MED14 reduced CDK8 and RNA polymerase II (RNAPII) recruitment, RNAPII C-terminal domain phosphorylation and impaired activation of IEG transcription. A single alanine substitution at S986 reduced activation of an E26 (ETS)-responsive reporter by oncogenic Ras and mitogen-induced, Elk-1-dependent transcription, whereas activities of other transcriptional activators were unaffected. We also demonstrate that Elk-1 can associate with MED14 independently of MED23, which may facilitate phosphorylation of MED14 by ERK to impart a positive and selective impact on mitogen-responsive gene expression.

## INTRODUCTION

The specific and temporal co-ordination of gene expression is a fundamental process of cell-based life with patterns of gene expression controlling proliferation, differentiation and cell death. Immediate early gene (IEG) expression has revealed how crucial regulatory events revolve around the interface between pathway-specific transcription factors and components of the transcription machinery, involving a plethora of protein interactions and modifications (1,2).

The ternary complex factor (TCF) Elk-1, an E26 (ETS) transcription factor family member, activates transcription of multiple IEGs in response to mitogens, a process initiated upon Elk-1 phosphorylation by the mitogen-activated protein kinases (MAPKs) extracellular signal-regulated kinase (ERK)1 and ERK2 (ERK) (3–5). Phosphorylation increases the affinity of Elk-1 for Serum Response Element (SRE)-containing promoters, which it engages in a complex with Serum Response Factor (SRF) (6), and correlates with desumoylation and derepression of Elk-1 to unleash its trans-activation potential (7,8). One means by which Elk-1 stimulates IEG expression is through the recruitment of histone acetyltransferases and concomitant alterations in nucleosome positioning, presumably facilitating promoter access and the establishment of pre-initiation complexes (PICs) (9).

RNA polymerase II (RNAPII) recognizes and engages promoters with the assistance of several basal transcription factors and accompanied by a multi-protein complex referred to as Mediator (10). Biochemical and structural analyses have revealed the mammalian Mediator to consist of up to 30 subunits with a mass of ∼1.5 MD in a head-body-tail arrangement plus auxiliary Cyclin-Dependent Kinase 8 (CDK8) module (11,12). In conjunction with regulatory transcription factors, Mediator appears to co-ordinate phospho-isomerization of the carboxy-terminal domain (CTD) of RNAPII that accompanies promoter escape and the switch to elongation (13). The role of the CDK8 module in these events is somewhat controversial, as evidence supports a repressor function at the level of initiation (14) but a stimulatory role during elongation (15). Thus the relationship between regulatory transcription factors, Mediator and the CDK8 module has become a major focus of studies on transcriptional regulation (16).

Elk-1 appears to communicate with Mediator via interactions with the MED23 subunit (aka Sur2, CRSP130, DRIP130) (17). This interaction appears dependent on phosphorylation of Elk-1 and is essential for Elk-1-dependent gene regulation in several contexts, including insulin-dependent adipocyte differentiation (18). What remains unclear is the nature of the interaction and its effect on the function of MED23 or indeed Mediator itself. We have shown that ERK is recruited to the promoters of IEGs by Elk-1 and inferred that ERK may phosphorylate additional substrates in PICs assembled on IEG promoters (19). Here we reveal the Mediator subunit MED14 as a novel ERK substrate, detect Elk-1 interactions with MED14 that are independent of MED23, demonstrate inducible phosphorylation of MED14 at IEG promoters and provide evidence for its positive role in mitogen-responsive gene transcription.

## MATERIALS AND METHODS

### Cell culture, transfections and extract preparation

HEK293, HEK293T, HeLa and NIH3T3 cells were cultured in Dulbecco’s modified Eagle's medium (DMEM) supplemented with 10% fetal calf serum (FCS), 2 mM l-glutamine, 100 U ml^−^^1^ penicillin and 100 µg ml^-1^ streptomycin. HCT116 cells were cultured in McCoy’s 5A medium supplemented with 10% FCS (Hyclone) and antibiotic/antimycotic mix (Gibco). Wild type and MED23^−^/^−^ Murine Embryonic Fibroblasts (MEFs) were cultured in DMEM (Sigma D5671) supplemented with 15% FCS, 4 mM l-glutamine, 100 U ml^−^^1^ penicillin and 100 µg ml^-1^ streptomycin. Cells were transfected according to standard procedures. Whole-cell lysates were prepared in RIPA buffer; nuclear and cytoplasmic extracts were prepared as described elsewhere (20).

Elk-1 knockdown was performed as described previously (19). For MED14 knockdown in HeLa cells a combination of two Silencer Select siRNAs (Ambion) were transfected with siPORT reagent according to the manufacturer’s instructions. Lentiviral knockdown in HCT116 cells was performed as described previously (15).

### Antibodies, immunopreciptations and immunoblotting

The antibodies used in this study are listed in Supplementary Table S1. The rabbit anti-phospho-S986 polyclonal antibody was raised against a phospho-peptide coupled to keyhole limpet haemocyanin and affinity purified with peptides coupled to sepharose beads. Co-immunoprecipitations and immunoblotting were carried out as detailed in Supplementary Information.

### Plasmids

The expression vectors used in this study are listed in Supplementary Table S2. Vectors for MED14Δ and GST-MED14-SPR were generated by standard procedures; point mutagenesis was by a polymerase chain reaction (PCR)-based protocol (QuickChange, Stratagene).

### *In vitro* kinase assays

Immobilized PICs, prepared as described earlier (19), were incubated in transcription buffer containing ^32^P-γ-ATP. Glutathione-S-transferase (GST) fusion proteins (1 µg) on sepharose beads or FLAG-/HA-tagged Mediator subunits immunoprecipitated from lysates of HEK293/293 T cells were incubated with active recombinant ERK2 (rERK2*) (0.25 µg) and ^32^P-γ-ATP as described earlier (19). ERK inhibitors I and II (Calbiochem) were used at 30 and 8.5 µM; Roscovitine was used at 30 µM.

### Quantitative reverse transcriptase PCR

The primers used in this study are listed in Supplementary Table S3. Assays were performed as described in the supplementary information.

### Reporter assays

Cells in 24-well plates were lysed 36 h after transfection in passive lysis buffer; Firefly and Renilla levels were analyzed with the Stop and Glow system (Invitrogen).

### Chromatin immunoprecipitation assays

The primers used in this study, methods and data analysis have all been described previously (15).

## RESULTS

### Identification of potential ERK targets in mediator

**Figure 1.**
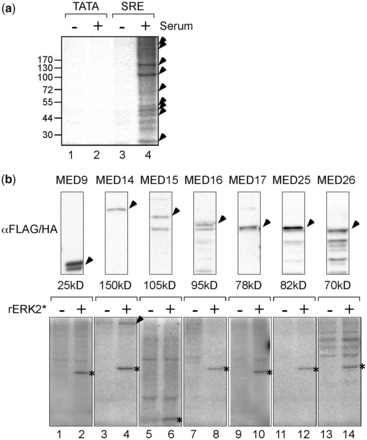
Phosphorylation of PIC components and Mediator subunits *in vitro* (**a**) Nuclear extracts from unstimulated (lanes 1 and 3) or serum-stimulated (lanes 2 and 4) HeLa cells were used for PIC assembly on TATA (lanes 1 and 2) or SRE (lanes 3 and 4) templates as described earlier (19). Complexes were washed, incubated in transcription buffer containing ^32^P-γ-ATP and resolved by SDS-PAGE. Gels were dried and visualized by phosphor-imaging (Fujifilm). Arrowheads indicate discrete radiolabeled species observed in multiple independent experiments. (**b**) Epitope-tagged Mediator subunits were expressed in HEK293 cells, immunoprecipitated from cell lysates with FLAG or HA antibodies (arrowheads, upper panels) and subjected to *in vitro* kinase assays with ^32^P-γ-ATP in the absence (−) or presence (+) of rERK2*. Reactions were separated by SDS-PAGE and visualized by phosphor-imaging (lower panels). Bands corresponding to auto-phosphorylated rERK2* are indicated by asterisks.

Our earlier report on the recruitment of kinases to IEG promoters described the presence of ERK in immobilized PICs assembled *in vitro* on SRE promoters (19). In subsequent kinase assays, we reproducibly detected phosphorylation of multiple proteins in PICs assembled on SRE promoters in nuclear extracts from mitogen stimulated cells (Figure 1a, lane 4) but not on basal promoters or in nuclear extracts from mitogen starved cells (lanes 1–3). Unfortunately, we were unable to ascribe any of these phosphorylation events unambiguously to ERK (unpublished data). However, an alternative candidate approach did allow us to identify ERK substrates at IEG promoters.

Several Mediator components had been identified in SRE PICs and some appeared to be good candidate ERK substrates, based on the presence of consensus ERK phosphorylation sites and docking motifs (see Table 1). To test these proteins as ERK substrates we immunoprecipitated tagged versions from cells for use in kinase assays with rERK2*. Of the seven proteins expressed successfully in HEK293 cells, only MED14 was phosphorylated in an ERK-dependent manner (Figure 1b, lower panels, lane 4). In further experiments, we confirmed this result and, in addition, demonstrated phosphorylation of MED1 and, weakly, MED15 (Supplementary Figure S1a lanes 6, 2 and 8). The lack of a suitable expression vector precluded MED13 from these analyses.

**Table 1.** Human mediator subunits with consensus ERK motifs

Rank	Subunit	MW	SP/ TP	PX_1/2_ (S/T)P	DEF	Phosphorylation	Module^a^
1	MED13	250	38	13	2	Yes^b^	cdk
2	MED1	220	41	7	0	Yes^c^	body
3	MED14	150	24	7	0	Yes	tail
4	MED15	105	16	6	0	ND	tail
5	MED26	70	17	5	0	Not ERK	unassigned
6	MED12	240	15	3	0	Yes	cdk
7	MED16	95	10	2	0	ND	tail
8	MED9	25	4	2	0	ND	body
9	MED25	82	5	1	0	ND	unassigned
10	MED17	78	3	1	0	ND	head

^a^based on (21), ^b^MDG and PES, unpublished, ^c^(22) and this work.

MED1 is a known ERK target (22) implicated together with MED14 in differential gene regulation by the glucocorticoid receptor (23). The ERK substrate apparent in MED12 immunoprecipitates (Supplementary Figure S1a, lane 4, open arrowhead) is substantially smaller than MED12 (240 kD) and could be a co-immunoprecipitated protein or a fragment of MED12. MED14 has a similar migration (see lane 6) and has been shown to co-purify with the CDK8 complex, of which MED12 is a subunit (14). However, although both MED12 and MED14 co-immunoprecipitate with CDK8 (Supplementary Figure S1b), we did not detect MED14 in MED12 immunoprecipitates, and the identity of this protein species remains open. We therefore focussed on the unreported relationship between ERK and MED14.

### Phosphorylation of MED14 by ERK *in vitro*

**Figure 2.**
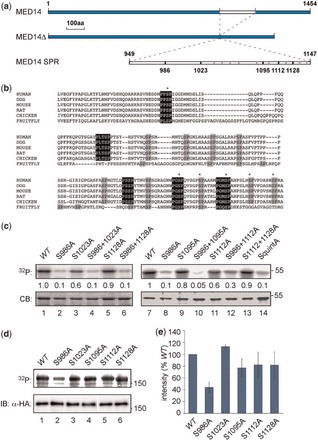
Mapping ERK phosphorylation sites in MED14. (**a**) Diagram showing location of SPR domain in MED14, the MED14Δ deletion mutant and the SPR domain of MED14 expressed as a recombinant protein. Black bars indicate ERK consensus sites screened by mutagenesis; gray bars indicate other non-consensus S/TP motifs. (**b**) Alignment of hMED14 SPR domain with MED14 amino acid sequences from HomoloGene group 22082 using the MUSCLE algorithm. MAPK consensus sites are highlighted in black with phosphorylated SP motifs identified by phospho-proteomics marked with asterisks. NCBI accession numbers for the MED14 proteins compared are: NP_004220.2, *Homo sapiens*; XP_538001.2, *Canis familiaris*; NP_001041673.1 *Mus musculus*; XP_228713.3, *Rattus norvegicus*; XP_416776.2, *Gallus gallus*; NP_612041.2, *Drosophila melanogaster*. (**c**) GST-MED14 SPR and the point mutants indicated were captured on glutathione-agarose beads and subjected to *in vitro* ERK kinase assays with ^32^P-γ-ATP. Reactions were separated by SDS-PAGE and visualized by phosphor-imaging (upper panels) and Coomassie blue staining (lower panels). Values indicate phosphate incorporation relative to wild-type (*WT*) fusion protein. (**d**) Full-length HA-tagged MED14 and the point mutants indicated were immunoprecipitated from HEK293T cell lysates and subjected to *in vitro* kinase assays with rERK2* and ^32^P-γ-ATP. Reactions were separated by SDS-PAGE and visualized by phosphor-imaging (upper panel, 80% of IP). Relative recoveries were assessed by immunoblotting for HA (lower panel, 20% of IP). (**e**) Phosphate incorporation into MED14 point mutants assayed as in (d) relative to wild-type (*WT*) protein (*n* = 3, error bars ± SD).

The carboxy-terminal half of MED14 includes an ∼200 residue region (aas 949–1147), with no obvious structural clues but a series of serine-proline (SP) motifs including several ERK consensus phosphorylation sites, that is conserved among vertebrates and flies (Figure 2a and b). Proteomic studies have identified several MED14 phospho-peptides derived from this region (24–26). To map potential ERK phosphorylation sites in MED14, this SP-rich region (SPR domain) was first expressed as a recombinant GST fusion and tested as an ERK substrate, then mutated to remove individual or multiple sites. ERK phosphorylated the SPR domain efficiently (Supplementary Figure S2a and Figure 2c, lanes 1 and 7). A single alanine substitution at S986 reduced phosphate incorporation by 90% (Figure 2c, compare lanes 1 and 2, 7 and 8), whereas mutations S1023A, S1128A, S1095A and S1112A (lanes 3, 5, 9 and 11) had little effect. Moreover, in combination with S986A only S1095A caused any further decrease in phosphorylation (compare lanes 8 and 10), suggesting that S986 is the predominant site for ERK phosphorylation within this region of MED14.

In kinase assays with MED14 proteins immunoprecipitated from HEK293T cells, full-length MED14 was phosphorylated efficiently by ERK but MED14 lacking the SPR (MED14Δ) was not (Supplementary Figure S2b), confirming that ERK phosphorylation was confined to the SPR domain. Single point mutations were introduced into full-length MED14 at the same five sites and, as seen with the recombinant SPR domain, S986A had the most significant effect, reducing ERK phosphorylation of MED14 by 60% (Figure 2d, lane 2 and e). Substitutions at other sites in combination with S986A did not further reduce MED14 phosphorylation (data not shown). Taken together, the data with the recombinant SPR domain and the entire protein indicate that S986 is the primary, but not the sole, ERK phosphorylation site in MED14.

The identification of additional MED14 SPR domain phospho-peptides besides those encompassing S986 suggests that the domain is a substrate for multiple kinases. As such motifs are often CDK targets and Mediator reversibly associates with the CDK8 complex, we considered that CDK8 might also phosphorylate the MED14 SPR domain. Co-immunoprecipitations with endogenous and ectopically expressed proteins confirmed that CDK8 and MED14 interact (Supplementary Figure S2c and d). We also detected MED12 (Supplementary Figure S1b) and MED23 (Supplementary Figure S2c) in CDK8 co-immunoprecipitates, which could conceivably influence kinase activity. However, CDK8 immunoprecipitated either directly or indirectly with overexpressed MED12 or MED14, failed to phosphorylate the recombinant SPR domain of MED14, with or without parallel phosphorylation by rERK2* (Supplementary Figure S2e). We therefore concluded that the SPR domain of MED14 is unlikely to be a substrate for CDK8.

### Phosphorylation of MED14 by ERK *in vivo*

**Figure 3.**
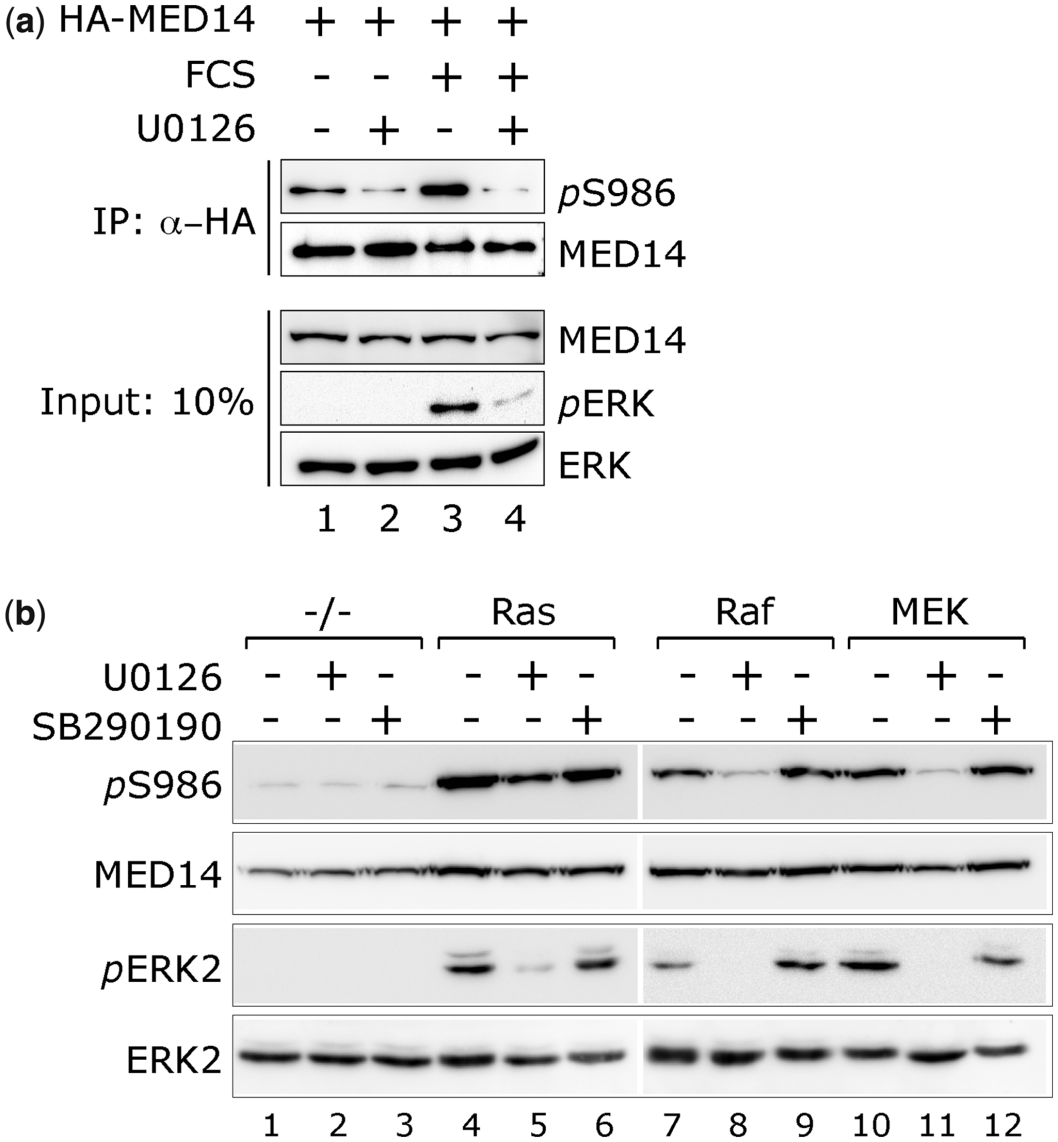
ERK phosphorylates MED14 S986 in response to mitogenic signals. (**a**) HeLa cells transfected with HA-MED14 were serum starved for 16 h, treated with the MEK inhibitor U0126 (+) or a DMSO control (−) for 2 h and harvested before (−) or after stimulation (+) with 10% FCS for 15 min. MED14 was recovered by immunoprecipitation from lysates (90% anti-HA, upper panel) and analyzed for S986 phosphorylation with a phospho-specific antibody. MED14 expression levels and ERK activation levels were confirmed by immunoblotting cell lysates (10%, lower panel). (**b**) HEK293 cells transfected with HA-MED14 alone (1–3) or with active alleles of Ras (4–6), Raf-1 (7–9) or MEK (10–12) were serum starved for 16 h and treated with the MEK inhibitor U0126, the p38 inhibitor SB290190 or DMSO control for 2 h. MED14 expression, S986 phosphorylation and ERK activation were monitored by immunoblotting cell lysates.

To determine if S986 in MED14 is phosphorylated by ERK *in vivo*, a phospho-specific antibody was developed. In MED14, immunoprecipitates from HeLa cells, the anti-*p*S986 antibody detected increased phosphorylation of MED14 in response to serum stimulation that was abolished by prior treatment of cells with the MAPK/ERK Kinase (MEK) inhibitor U0126 (Figure 3a). To confirm that ERK was responsible for MED14 phosphorylation, we used mutants of the Ras/ERK cascade. Activated versions of Ras, Raf and MEK all stimulated phosphorylation of MED14 and ERK, which could be substantially blocked by U0126 but not by the p38 inhibitor SB290190 (Figure 3b). In contrast, active versions of Phosphotidylinositol-3OH-kinase (PI3K), Akt/PKB, the small G proteins RhoA, Rac1 and cdc42 and their downstream kinases SEK and PAK activated ERK weakly at best and did not stimulate MED14 phosphorylation (Supplementary Figure S3). Expression of a truncated active version of MEKK1 (27) efficiently induced MEK-dependent phosphorylation of S986 and also ERK (Supplementary Figure S3, lane 3). Given the correlation between ERK activation and MED14 phosphorylation in these experiments, we conclude that S986 in MED14 is a *bona fide* target for ERK and mitogen signaling. Importantly, the experiments do not rule out other sites in the SPR domain as targets for alternative proline-directed kinases activated by these pathways.

### MED14 is required for efficient IEG transcription

**Figure 4.**
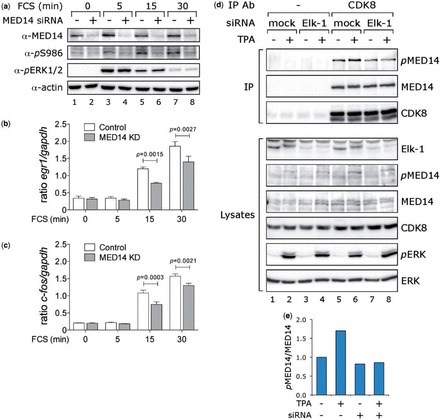
MED14 knockdown impairs IEG induction. Hela cells were mock transfected (−) or transfected twice with two MED14-targetting siRNAs (+). After recovery for 24 h, serum was withdrawn for 16 h, cells were then stimulated for 0, 5, 15 or 30 min with FCS and harvested for protein and RNA extraction. (**a**) Analysis of MED14 knockdown, S986 phosphorylation and ERK activation by immunoblotting with the antibodies indicated. (**b** and **c**) Analysis of MED14 knockdown on c-*fos* and *egr1* RNA expression following serum stimulation by quantitative reverse transcriptase PCR. Data represent mean values from three experiments each performed in triplicate. Multiple two-tailed t-tests (assuming equal SDs, with Holm-Sidak correction using GraphPad Prism 6) were carried out to compare means (control versus MED14 KD) at each time point (*n* = 3, *P* < 0.001 at 15 min and 30 min for both c-*fos* and *Egr1*). (**d**) HeLa cells were mock transfected or transfected with Elk-1 siRNA, then serum-starved (−) or starved and stimulated with TPA for 15 min (+). CDK8 immunoprecipitates were collected from whole-cell lysates and the presence and phosphorylation of MED14 was determined by SDS-PAGE and immunoblotting with the antibodies indicated (upper three panels). Lower panels of lysates indicate extent of Elk-1 knockdown (∼70%) and of ERK activation (*p*ERK). (**e**) Quantification of *p*MED14/MED14 ratios in the experiment shown in (d).

As phosphorylation of S986 in MED14 occurs in response to mitogen or oncogene stimulation of ERK, we next determined the contribution of MED14 to the regulation of mitogen-responsive genes. First we tested the effect of siRNA-mediated knockdown of MED14 in HeLa cells on serum induction of several IEGs. Endogenous MED14 knockdown was achieved with a combination of two MED14 siRNAs (Figure 4a, upper panel), with concomitant loss of signal detected by the *p*S986 antibody (second panel), which followed the activation of ERK induced by serum (third panel). Parallel analysis of gene expression by quantitative reverse transcriptase PCR revealed that MED14 knockdown reduced the induction of c-*fos* RNA by 50% at 15 min (Figure 4b) and had a similar effect on *Egr1* induction (Figure 4c). MED14 knockdown did not affect the expression of *Mcl1*, which in these cells was relatively high and unresponsive to serum stimulation (Supplementary Figure S4), indicating that MED14 depletion does not inactivate Mediator *per se*.

If Elk-1 is required to recruit ERK for mitogen-induced *p*S986 phosphorylation, loss of Elk-1 might abrogate this modification to MED14. We therefore examined the effect of siRNA-mediated Elk-1 knockdown on the phosphorylation of *p*S986 in Mediator complexes isolated from HeLa cells by immunoprecipitation with a CDK8 antibody. Phosphorylation of MED14 in CDK8-Mediator complexes was apparent in unstimulated cells (upper panel, Figure 4d, lane 5) and increased upon mitogen stimulation (lane 6 and Figure 4e). Elk-1 knockdown (∼70%) reduced basal phosphorylation only slightly, but prevented mitogen-induced phosphorylation of MED14 (compare lanes 7 and 8). Thus, Elk-1 participates in ERK phosphorylation of MED14 in CDK8-Mediator complexes.

To assess the contribution of MED14 phosphorylation by ERK towards IEG induction, we attempted to rescue the effect of MED14 knockdown with native and mutant versions of siRNA-resistant MED14 expressed transiently in HeLa cells from plasmids or in HCT116 cells from lentiviral vectors (15). However, responses of HeLa cells transfected with both siRNA and MED14 expression vectors to mitogens were erratic and rescue attempts in cells transduced with lentivirus were unsuccessful. Nonetheless, the knockdown experiments confirm a role for MED14 in mitogen induction of IEGs, albeit with the caveat that MED14 depletion may have pleiotropic effects on Mediator structure and composition.

### Mutation of S986 impairs oncogene and mitogen-induced transcription

**Figure 5.**
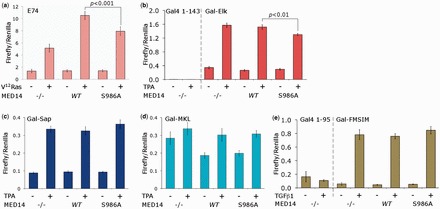
S986 mutation impairs Ras- and mitogen-induced reporter expression. (**a**) HEK293 cells were transfected with an E74-Luc reporter and TK-Renilla control, expression vectors for MED14, MED14-S986A or corresponding vector (pRK5) and V^12^-Ras or corresponding vector (pCMV5). After 36 h cell gene expression was analyzed by fluorescence assay. Data represent mean values from three independent experiments each performed in triplicate (*P* < 0.001). (**b–d**) NIH3T3 cells were transfected with a Gal4-Luc reporter and UbC-Renilla control, expression vectors for MED14, MED14-S986A or corresponding vector (pRK5) and Gal4(1–143) or Gal4-protein fusions with activation domains of Elk-1, Sap1a or MKL1, respectively. After serum-starvation for 24 h, cells were left untreated (−) or stimulated with TPA for 6 h (+). Data in (b) represent mean values from four experiments, in (c) and (d) from three experiments, all performed in triplicate. (**e**) NIH3T3 cells were transfected with a Gal4-Luc reporter and UbC-Renilla control, expression vectors for MED14, MED14-S986A or corresponding vector (pRK5) and Gal4(1–95) or a Gal4 protein fusion with FM/SIM SMAD recruitment motifs. After serum-starvation for 24 h, cells were left untreated (−) or stimulated with TGFβ for 6 h (+). Data represent mean values from three experiments each performed in triplicate.

Given the inconclusive outcome from RNAi knockdown and rescue experiments in assessing the contribution of MED14 phosphorylation to IEG activation we turned to gene reporter assays. First we transfected a Ras-responsive reporter gene carrying multimerized ETS binding sites, to which Elk-1 and other ETS factors bind (28), into HEK293 cells. Expression of MED14 doubled the transcriptional response to active Ras and this effect was significantly impaired by the S986A substitution (Figure 5a).

To focus specifically on Elk-1-dependent gene expression in response to mitogens we turned to a Gal4 reporter and the well-documented Elk-1 fusion protein consisting of the Gal4 DNA binding domain in place of the ETS domain (Gal-Elk) (18,29). Initial experiments were inconclusive because MED14 expression influenced both reporter and control genes. However, in NIH3T3 cells we were able to achieve comparatively stable control gene expression by switching promoters (see Supplementary Figure S5a), whereupon MED14-S986A significantly reduced mitogen-induced reporter expression when compared with native MED14 (Figure 5b). This effect is apparent even though endogenous MED14 is present in the cells. Western blots confirmed that expression levels of MED14 and MED14-S986A were indistinguishable in both HEK293 and NIH3T3 cells (Supplementary Figure S5b).

The observation that ectopic expression of a core component of Mediator was able to influence a control gene led us to explore whether MED14 expression and its phosphorylation on S986 had a generic influence on multiple transcriptional activators. Elk-1 is one of three TCFs, the others being Net (Elk-3) and Sap1 (Elk-4) (3,4). Like Elk-1, when Sap1 is fused to Gal4 (Gal-Sap) it also mediates induction of Gal4 reporter expression in response to mitogens. In this case, however, expression of MED14-S986A did not impair reporter expression (Figure 5c).

At some promoters, Elk-1 forms ternary complexes with SRF in competition with Myocardin-like factors. A Gal4 fusion containing the activation domain of MKL1 (507–931) (30) was also active, but only weakly responsive to 12-O-tetradecanoylphorbol-13-Acetate (TPA), and although MED14 expression reduced basal expression, there was no difference between native MED14 and MED14-S986A (Figure 5d). Other studies describe crosstalk between ERK and transforming growth factor beta (TGFβ) signaling (31,32). We therefore also tested SMAD2-dependent gene expression using a Gal4 fusion with the CTD of XfoxH1b, which carries Fast/FoxH1 and SMAD interacting motifs (33). Again, ectopic expression of either MED14 or the S986A mutant had no significant effect on TGFβ-induced reporter expression (Figure 5e).

Taken together, these data indicate that ERK phosphorylation of MED14 contributes positively to mitogen-induced gene expression mediated by Elk-1 and possibly by other ETS factors. Moreover, this effect is selective, as the activity of several other transcriptional activators is unimpaired by the S986A substitution in MED14.

### MED14 phosphorylation induced at IEG promoters

**Figure 6.**
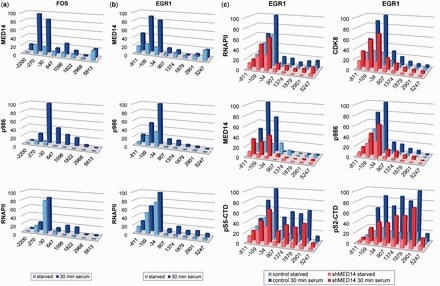
MED14 phosphorylation at IEG promoters. (**a**) ChIP analysis of MED14 recruitment (upper graph), S986 phosphorylation (middle) and RNAPII occupation (bottom) at the c-*fos* gene in starved (light blue) or serum stimulated (dark blue) HCT116 cells. Data represent mean values from three experiments as mean percentage of maximal signal at each locus (in ng of input DNA). (**b**) ChIP analysis as in (a) of MED14 recruitment (upper graph) S986 phosphorylation (middle) and RNAPII occupation (bottom) at the *Egr1* gene. Data represent mean values from four experiments as mean percentage of maximal signal at each locus (in ng of input DNA). (**c**) HCT116 cells expressing MED14 shRNA or control cells were serum starved or starved and treated with serum for 30 min. RNAPII occupation and CDK8 recruitment (upper graphs), MED14 recruitment and S986 phosphorylation (middle), pS5-CTD and pS2-CTD modifications (bottom) at the *Egr1* gene were determined by ChIP assay. Data represent mean values from three experiments as mean percentage of maximal signal at each locus (in ng of input DNA).

The recruitment of active ERK by Elk-1 to mitogen-responsive gene promoters implicates them as locations at which phosphorylation of MED14 may occur. In chromatin immunoprecipitation (ChIP) experiments with HCT116 cells, mitogens stimulated the recruitment of MED14 to the c-*fos* promoter region (Figure 6a), in line with observations for other Mediator components (19,34). Mediator recruitment was accompanied by a substantial increase in MED14 phosphorylation on S986. Little change in promoter occupancy by RNAPII was observed, in agreement with previous reports (15), but whereas RNAPII was restricted to the TATA box region in unstimulated cells, its presence on the gene was apparent after stimulation. As anticipated, similar patterns of occupation and phosphorylation were observed at the *Egr1* promoter (Figure 6b). Thus, phosphorylation of MED14 on S986 at the promoters of these two IEGs accompanies their transcriptional activation by mitogens.

Using lentiviral-mediated knockdown we were able to reduce MED14 levels at the *Egr1* and c-*fos* promoters by ∼50% in serum-stimulated cells (Figure 6c and Supplementary Figure S6) with concomitant decreases in S986 phosphorylation. MED14 knockdown was associated with reductions in RNAPII occupation and CDK8 recruitment within the timeframe of mitogen stimulation. In addition, we observed decreased phosphorylation of the CTD, in the case of phospho-serine 5 at the promoter and for phospho-serine 2 also along the genes (Figure 6c and Supplementary Figure S6). These reductions are consistent with the impairment of c-*fos* and *Egr1* transcription seen with MED14 siRNA (Figure 4b and c). Taken together, these data confirm the participation of MED14 and its phosphorylation on S986 in the transcriptional activation of the c-*fos* and *Egr1* genes in response to mitogens.

### Elk-1 interactions with MED14

**Figure 7.**
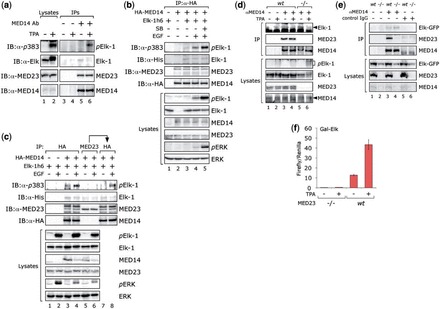
Elk-1 interacts with MED14 independently of MED23. (**a**) Lysates were prepared from serum-starved (−) or TPA-stimulated (+) HeLa cells (lanes 1 and 2). Control (lanes 3 and 4) or MED14 (lanes 5 and 6) co-immunoprecipitates were collected and analyzed in parallel by SDS-PAGE and immunoblotting for the presence of phospho-Elk (upper), Elk-1, MED23 and MED14 (lower panel) with the antibodies indicated. (**b**) HEK293 cells were transfected with vectors for tagged versions of MED14 and Elk-1 as indicated. Cells were serum-starved (lanes 1–3), treated with EGF alone (lane 4) or with EGF after prior addition of the p38^mapk^ inhibitor SB290190 (lane 5). Lysates were prepared, MED14 co-immunoprecipitates were collected with an anti-HA antibody and analyzed by SDS-PAGE and immunoblotting for the presence of phospho-Elk (upper), Elk-1, MED23 and MED14 (lower panel) with the antibodies indicated left. Immunoblots of corresponding lysates are shown in the panels below. (**c**) HEK293 cells were transfected with vectors for tagged versions of MED14 and Elk-1 as indicated. Lysates were prepared from serum-starved cells (lanes 1, 3, 5 and 7) or cells treated with EGF (lanes 2, 4, 6 and 8). Co-immunoprecipitates were collected from the lysates with an anti-HA antibody (lanes 1–4), an anti-MED23 antibody (lanes 5 and 6) or with an anti-HA antibody from the post MED23 IP lysates (lanes 7 and 8). Samples were analyzed by SDS-PAGE and immunoblotting for the presence of phospho-Elk (upper), Elk-1, MED23 and MED14 (lower panel) with the antibodies indicated left. Upper bands in lanes 3, 4, 7 and 8 of MED23 panel are from previous exposure to MED14 antibody. Immunoblots of corresponding lysates are shown in the panels below. (**d**) MED23 *wt* (lanes 1–4) and MED23−/− MEFs (lanes 5 and 6) were transfected with a vector for Elk-1, and lysates were prepared from serum-starved cells (lanes 1, 3 and 5) or cells treated with TPA (lanes 2, 4 and 6). Mock (lanes 1 and 2) or MED14 co-immunoprecipitates (lanes 3–6) were collected from the lysates and samples were analyzed by SDS-PAGE and immunoblotting for the presence of Elk-1 (upper), MED23 (middle) and MED14 (lower panel). Immunoblots of corresponding lysates are shown in the panels below. (**e**) Lysates were prepared from MED23 *wt* (lanes 1, 3 and 5) and MED23−/− MEFs (lanes 2, 4 and 6) transfected with a vector for Elk-GFP. Mock (lanes 1 and 2), MED14 (lanes 3 and 4) or control IgG co-immunoprecipitates (lanes 5 and 6) were collected from the lysates, and samples were analyzed by SDS-PAGE and immunoblotting for the presence of Elk-GFP (upper), MED23 (middle) and MED14 (lower panel). Immunoblots of corresponding lysates are shown in the panels below. (**f**) MED23−/− and MED23 *wt* MEFs were transfected with Gal4-Luc reporter, Gal-Elk expression vector and UbC-Renilla control. After serum-starvation for 24 h, cells were left untreated (−) or stimulated with TPA for 6 h (+). Data represent mean values from three experiments each performed in triplicate.

Elk-1 is thought to contact Mediator through phosphorylation-dependent interactions with MED23 (17). As MED23 co-immunoprecipitates efficiently with MED14 and CDK8 (see e.g. Supplementary Figure S2c), we reasoned that an indirect interaction between Elk-1 and MED14 might also be detectable. Indeed, in MED14 co-immunoprecipitates from HeLa cells, we observed MED23 and also Elk-1 (Figure 7a, lane 5). In co-immunoprecipitates from mitogen-stimulated cells, we also detected phospho-Elk-1 (lane 6). These findings could be recapitulated in HEK293T cells expressing tagged versions of MED14 and Elk-1 (Figure 7b and Supplementary Figure S7a), phosphorylated Elk-1 only being present after mitogen stimulation and more apparent when ERK signaling was enhanced with the p38 inhibitor SB290190 (Figure 7b, lane 5). Nonetheless, the amount of Elk-1 was similar under all conditions. These results indicate that a fraction of Elk-1 contacts Mediator regardless of gene activation state, which is consistent with the detection of Elk-1 and several Mediator subunits at IEG promoters in unstimulated cells (19).

The data above suggest that Elk-1-Mediator interactions involving MED23 may not be contingent on Elk-1 phosphorylation. To assess this we performed MED14 and MED23 co-immunoprecipitations in parallel. Elk-1 was present in both MED14 and MED23 co-immunoprecipitates (Figure 7c, lanes 3–6). However, phospho-Elk-1 was present in MED14 but not in MED23 co-immunoprecipitates collected from mitogen-stimulated cells (compare lanes 4 and 6). To explain this result, we surmised that the MED23 antibody preferentially recognizes a Mediator-independent population of MED23. Indeed, when MED14 was immunoprecipitated from cell lysates after immunoprecipitation of MED23, Elk-1, phospho-Elk-1 and MED23 were all detected (lanes 7 and 8). This result indicates that phospho-Elk-1 only interacts with MED23 in a complex with MED14.

If the observed association between MED14 and Elk-1 were dependent on MED23, Elk-1 would unlikely be present in MED14 co-immunoprecipitates from cells lacking MED23. To test this we performed experiments with MED23*wt* and MED23−/− MEFs. In fact the association of MED14 and Elk-1 was equally apparent in MED23*wt* and MED23−/− MEFs, even though expression of Elk-1 was somewhat lower in MED23−/− MEFs (Figure 7d, compare lanes 3 and 5). This result was confirmed with an Elk-GFP fusion protein (Figure 7e). Thus without MED14, MED23 does not contact phospho-Elk, whereas Elk-1 is able to contact Mediator independently of MED23. Moreover, MED14 phosphorylation increases following mitogen stimulation in both MED23*wt* and MED23−/− MEFs (Supplementary Figure S7b). Nonetheless, Gal-Elk fails to activate reporter expression in response to mitogens in MED23−/− cells (Figure 7f), consistent with MED23 acting in a post-recruitment step (34).

## DISCUSSION

Our studies have revealed that active ERK is recruited by Elk-1 into PICs at mitogen-responsive promoters and phosphorylates MED14 on S986. This phosphorylation event at IEG promoters contributes to transcriptional activation, as an alanine substitution at S986 causes significant reductions in Ras-induced and mitogen-stimulated gene expression. Phosphorylation of S986 by ERK may be facilitated by Elk-1 interactions with MED14, which are detected before mitogen stimulation and in cells lacking MED23. Our data are consistent with a role for MED23 in post-recruitment events that may include conformational changes to Mediator induced by phosphorylation of MED14.

### MED14 phosphorylation and structure

Serine 986 is one of 14 S/TP motifs defining a SP-rich (SPR) domain in MED14 that appears to undergo multiple phosphorylations by hitherto unidentified kinases. All bar one MED14 phospho-peptides identified to date in proteomic screens map to this region. Only 5 S/TP motifs (including S986) conform to the ERK consensus, of the others, three (S1119, S1136 and S1146) are phosphorylated in MV4-11, HCT116 and/or HEK293 cells, as are two adjacent serines (S1133 and S1144) (24–26). Our data establish ERK as the S986 kinase; they also rule out phosphorylation of the SPR domain by CDK8. Importantly, they do not exclude phosphorylation of sites other than S986 by alternative proline-directed kinases. Phosphorylation site prediction algorithms identify 21 high confidence sites, of which 12 are SP motifs. Other kinases predicted to phosphorylate the SPR domain include ATM and IKK (35,36). Thus the SPR domain in MED14 may serve as an integration point for multiple pathways that influence Mediator function.

Early genetic experiments in yeast complemented by fractionation and reconstitution studies predicted the modular nature of Mediator. EM studies later revealed a three-lobed structure with head, body and tail domains (12). MED14, the product of an essential gene in yeast (*Rgr1*), is a core structural component of the tail domain, acting as a scaffold for several submodules defined by MED9, MED10 and MED15 (Gal11). Genetic deletion of Gal11 in yeast yielded a Mediator complex lacking the entire Gal11 module and rendered RNAPII defective for activated but not basal transcription (37). Subsequent identification of Gal11 subunits (MED2, 3, 15 and 16) as co-activators for numerous mammalian transcription factors has reinforced the notion of the tail domain as the entry point for regulatory input to the RNAPII holoenzyme. In line with this model, loss of MED24 impairs the incorporation of MED23 into Mediator and vice versa (17,38).

Interpretations of interaction studies in yeast inferred that tail domain subunits of the Gal11 submodule (e.g. MED16) contact the C-terminal part of MED14, while regions of MED14 further N-terminal interact with MED9 and MED10 submodules, which form part of the body domain (39,40). Extrapolating from this organization to the metazoan counterpart, the SPR domain of MED14 could provide an articulation point between body and tail of Mediator. Although sequence comparisons provide no clues to domain structures in MED14 and structural data are presently unavailable, the regular distribution of S/TP motifs with a mean spacing of 8.5 and protein structure predictions suggest a domain with conformational flexibility. Nonetheless, recombinant versions of the MED14 SPR domain are soluble and structural studies may be informative.

### MED14 phosphorylation and IEG transcriptional activation

MED14 participates in hormone-responsive gene expression (41). Using RNAi we extended its role to include mitogen-responsive gene expression, with induced levels of c-*fos* and *Egr1* RNA being decreased ∼50% by a similar degree of MED14 knockdown. In contrast, *Mcl1* expression was unaltered, suggesting that this reduction was unlikely to result from a pleiotropic defect to Mediator. ChIP analyses further indicated that the lower levels of c-*fos* and *Egr1* RNA correlated with reductions in both CDK8 and RNAPII occupation at the two promoters and a decrease in pS2-CTD density along the genes.

Reporter gene assays provided evidence for a role of MED14 phosphorylation on S986 in IEG induction. Ras-dependent activation of an ETS-responsive reporter in HEK293 cells increased substantially on co-expression of MED14 but significantly less with the S986A mutant, implicating ERK phosphorylation of MED14 in proliferative gene expression. In addition, we were able to demonstrate a significant dominant-negative impact of the S986A point mutation on Elk-1 transactivation. Again this effect was specific, as no significant difference was observed with analogous reporters for Sap1a, MKL1 or SMAD2/3.

Phosphorylation of MED14 increased rapidly and substantially at the c-*fos* and *Egr1* promoters after mitogen stimulation (20 - and 4-fold after 30 min respectively), promoters to which Elk-1 recruits ERK (19). These earlier knockdown experiments have now been extended to highlight the role of Elk-1 in MED14 phosphorylation by ERK (Figure 4d). On the c-*fos* promoter phosphorylated MED14 was restricted to a peak at the region around −30 (Figure 6a), consistent with phosphorylation of MED14 within the recruited RNAPII holoenzyme. Phosphorylated MED14 also accumulated and co-localized with increases in CDK8 recruitment and S5-CTD phosphorylation (Figure 6c). These data are consistent with a model in which ERK phosphorylates MED14 within PICs as a positive post-recruitment step during IEG induction.

### Elk-1 interactions with MED14 and Mediator

MED23 co-immunoprecipitates efficiently with MED14, so the presence of Elk-1 in MED14 co-immunoprecipitates was anticipated, given the essential contribution of MED23 to the Elk-1-dependent activation of IEGs and the induction of adipogenesis (17,18). However, in contrast to published data, our findings with endogenous proteins in HeLa cells and tagged versions of MED14 and Elk-1 in HEK293T cells indicated that unphosphorylated and phosphorylated Elk-1 interact similarly with Mediator. Moreover, phospho-Elk-1 was co-immunoprecipitated with MED14, but not with MED23, while interactions between MED14 and Elk-1 could be detected equally well in MED23−/− and MED23 *wt* MEFs. To explain the first point we infer that the MED23 antibody immunoprecipitates a pool of free MED23 but is unable to recognize MED23 associated with Mediator. This may have a bearing on the interpretation of earlier work, although the implications of a free pool of MED23 are presently unclear. From the second, we conclude that MED23 is not essential for the interaction between Elk-1 and MED14, nor, apparently, for MED14 phosphorylation (Supplementary Figure S7b), and infer that the crucial role of MED23 is not as intermediary in the recruitment of Mediator by Elk-1.

Recently, Yin *et al.* showed that MED23−/− MEFs were predestined to a smooth muscle cell phenotype, concluding that the absence of MED23 allowed Myocardin-like factors to predominate over TCFs at common target gene promoters (42). Our data imply that in MED23−/− MEFs Elk-1 would not be precluded from PIC formation at such promoters but would be unable to stimulate post-recruitment events. This scenario is fully consistent with an earlier conclusion that MED23 serves its co-activator function during the post-recruitment phase of transcription (34).

MED14 has been linked to transcriptional regulation of hormone-responsive genes in conjunction with MED1. Two transactivation domains of the glucocorticoid receptor cooperate independently with MED1 and MED14 (23). Similarly, PPARγ, which was initially shown to require MED1, also interacts with MED14 via its N-terminal domain (43). At present we have no evidence to indicate that Elk-1 interacts directly with MED14. Submodules containing MED9, MED10 and MED16 all contact Mediator via MED14, providing several alternatives, although the absence of MED16 and MED24 from Mediator complexes in MED23−/− cells appears to exclude them as targets. However, the link between Elk-1 and MED23 is no less enigmatic. The Elk-1 activation domain was shown to bind HeLa Mediator in a phosphorylation-dependent manner, but the interaction was not clearly dependent on MED23 (17). Furthermore, no biochemical dissection of the MED23 interaction with Elk-1 has been forthcoming, whereas two *Drosophila* transcriptional activators could be shown to interact directly with a 270aa fragment of dMED23 (48% identical to hMED23) (44). In summary, by whatever means, Elk-1 communicates with Mediator, our finding that an interaction can be established independently of MED23 suggests that the documented association between Elk-1 and MED23 may ultimately prove to be crucial for post-recruitment operations involving Mediator.

### MED14 phosphorylation and Mediator function

How might MED14 phosphorylation promote IEG transcription? Mediator acts as a central scaffold for PIC assembly, interacting with its dedicated kinase module, the basal transcription machinery and RNAPII (12). Thus, modification of S986 may control accessibility to Mediator, by the creation or disruption of a critical docking site. For example, 14-3-3 proteins recognize a range of phospho-serine containing partners; conversely, phosphorylation of the GS- region of TGFβRI blocks binding by FKBP12 (45,46). We did not detect a change in the global association of CDK8 and MED14 upon mitogen stimulation of HeLa cells (accompanied by MED14 phosphorylation), possibly because such changes are likely to be dynamic and restricted to a small fraction of Mediator. Additionally, phosphorylation site mutations in MED14 had no impact on its association with CDK8 (LL and PES, unpublished). However, these experiments did not rule out other changes to CDK8-associated Mediator complexes.

The conformation of Mediator differs between forms bound to CDK8 and RNAPII (41) and it has been shown to undergo structural shifts triggered by interactions with the transactivation domain of p53 that correlated with RNAPII activation (47). Conceivably, phosphorylation offers a potent mechanism to influence Mediator conformation. Interestingly, the WW domain-containing protein Pin1 is an isomerase implicated in IEG induction that recognizes phospho-serine/threonines, allowing kinases and phosphatases to exploit conformational change around peptidyl-prolyl bonds (48).

Indirect evidence attests to a key role for the SPR domain in MED14 function. Deletion of the SPR domain from MED14 (MED14Δ) generated a mutant that no longer accumulated in the nucleus (unpublished), hence its exclusion from functional assays, suggesting that the SPR domain might be required for MED14 incorporation into Mediator. However, interaction studies in yeast suggest that the SPR domain nestles between regions of MED14 with which diverse submodules interact. It would thus be ideally positioned to engage in post-recruitment conformational changes to Mediator that could result from its phosphorylation by ERK and, potentially, several other kinases. Furthermore, MED14 is just one of multiple ERK targets within Mediator, as MED1 and at least one other Mediator subunit are phosphorylated by ERK (22) (and our unpublished data). ERK recruitment by different transcriptional activators or mechanisms (49) may dictate its target selection within Mediator. Alternatively, multiple contemporaneous phosphorylation events could have a synergistic effect on Mediator conformation unlikely to be abrogated by individual point mutations in a single Mediator subunit. Either way, our demonstration that ERK is recruited into PICs by the activation domain of Elk-1 to phosphorylate the core Mediator component MED14 and promote transcription highlights a new aspect to post-recruitment regulation of IEG expression.

## SUPPLEMENTARY DATA

Supplementary Data are available at NAR Online, including [50–52].

## FUNDING

The BBSRC [BB/D019117/1 to P.E.S.]; NIH [RO1CA117907 to J.M.E. and M.D.G.] and NSF [MCB-1243522]. Funding for open access: University of Nottingham.

*Conflict of interest statement*. None declared.

## Supplementary Material

Supplementary Data
