# Supplementary material for: ERK phosphorylation of MED14 in promoter complexes during mitogen-induced gene activation by Elk-1
Source: Nucleic Acids Res. 2013 Sep 17;41(22):10241–53. doi: 10.1093/nar/gkt837 (PMC3905876; doi:10.1093/nar/gkt837)
Supplement: Supplementary Data [file supp_41_22_10241__index.html]

ERK phosphorylation of MED14 in promoter complexes during mitogen-induced gene activation by Elk-1 — ERK phosphorylation of MED14 in promoter complexes during mitogen-induced gene activation by Elk-1 — Supplementary Data 

# ERK phosphorylation of MED14 in promoter complexes during mitogen-induced gene activation by Elk-1

## Supplementary Data

files

**Files in this Data Supplement:**

- Supplementary Data - doc file
