# Supplementary material for: ERK phosphorylation of MED14 in promoter complexes during mitogen-induced gene activation by Elk-1
Source: Nucleic Acids Res. 2013 Sep 17;41(22):10241–53. doi: 10.1093/nar/gkt837 (PMC3905876; doi:10.1093/nar/gkt837)

**SUPPLEMENTARY INFORMATION**

**Table S1. Antibodies**

| **ANTIBODY** | **CODE** | **SOURCE** |
| --- | --- | --- |
| Elk-1 | H160 | Santa Cruz |
| phospho-Elk (pS383) | B4 | Santa Cruz |
| FLAG | M2 | Sigma |
| HA | 3F10 | Roche |
| His | AD1.1.10 | ABD Serotec |
| ERK | C14 | Santa Cruz |
| phospho-ERK | E4 | Santa Cruz |
| Actin | A2066 | Sigma |
| RNAPII (Rpb1) | 8WG16 | Abcam |
| pS2-CTD | H5 | Abcam |
| pS5-CTD | H14 | Abcam |
| CDK8 | C19 | Santa Cruz |
| MED14 (CRSP2) | 72141 | Abcam |
| phospho-MED14 | pS986 | This work |
| MED23 (IP) | A300-424A | Bethyl |
| MED23 (Western) | 550429 | BD Biosciences |

**Table S2. Expression Plasmids**

| **PROTEIN** | **VECTOR** | **SOURCE** |
| --- | --- | --- |
| MED1 (TRAP220) | pCDNA3.1/Hyg | Joan Conaway (Stowers Inst. Kansas) |
| MED9 | pCDNA3.1/Hyg | Addgene |
| MED12 | pCDNA3.1/Hyg | Joan Conaway (Stowers Inst. Kansas) |
| MED14 (CRSP2) | pRK5 | Michael Garabedian (NYU School of Medicine) |
| MED15 | pCDNA3.1/Hyg | Joan Conaway (Stowers Inst. Kansas) |
| MED16 (TRAP95) | pCDNA3.1/Hyg | Joan Conaway (Stowers Inst. Kansas) |
| MED17 | pCDNA3.1/Hyg | Addgene |
| MED23 (Sur2) | pCS2 | Arnie Berk (MBI, UCLA) |
| MED25 | pCDNA3.1/Hyg | Joan Conaway (Stowers Inst. Kansas) |
| MED26 | pCDNA3.1/Hyg | Addgene |
| CDK8 | pIRESneo | Yoshiaki Ohkuma (Toyama) |
| V^12^Ras | pCMV5 | Melanie Cobb (UTSW, Dallas) |
| Raf-259D | pCMV5 | Walter Kölch (Dublin) |
| MEK-R4F | pMCL | Natalie Ahn (Colorado) |
| MEKK1 | pCMV5 | Reference |
| p110-CAAX | pCDNA3 | Mike Waterfield (London) |
| Akt-CA | pCMV6 | Mike Greenberg (Boston) |
| RhoA | pCMV5 | Melanie Cobb (UTSW, Dallas) |
| Rac1 | pCDNAIIIB | Silvio Gutkind (NIH, Bethesda) |
| cdc42 | pCDNAIIIB | Silvio Gutkind (NIH, Bethesda) |
| SEK-ED (p54^sapk^) | pMT2 | Jim Woodgett (Toronto) |
| PAK1 | pCMV5 | Melanie Cobb (UTSW, Dallas) |
| Elk-1 | pCMV5 | Reference |
| Elk-dbl | pCMV5 | Reference |
| Elk-GFP | pEGFP-N1 | Reference |
| Renilla | pGL4 hRluc TK | Promega |
| Renilla | pGL4 hRluc UbC | This work |
| Gal-Elk | pSG424 | Reference |
| Gal-Sap | pSG424 | Reference |
| Gal-MKL1 | pCGN | Ron Prywes (Columbia, NY) |
| Gal-FMSIM | pEFplink | Caroline Hill (CRI, London) |

Table S3. RT-PCR Probes and Primers

| **Gene** | **NCBI ID** | **Forward primer (5’-3’)** | **Reverse primer (5’-3’)** | **Taqman Probe (5’-3’)** |
| --- | --- | --- | --- | --- |
| *c-fos* | NM_00525.2 | ACTACCACTCACCCGCAGAC | GTGGGAATGAAGTTGGCACT | CCTGTCAACGCGCAGGACTTCTG |
| egr-1 | NM_001964.2 | CAGCACCTTCAACCCTCAG | CAGCACCTTCTCGTTGTTCA | CTACGAGCACCTGACCGCAGAGTCTT |
| *mcl-1* | NM_021960.3  NM_182763.1 | ACGGGTCACTACCCTCGAC | CCCATTGGCTTTGTGTCCT | TACCGGCAGTCGCTGGAGATTATCTC |
| gapdh | NM_002046.3 | CTGCACCACCAACTGCTTAG | ACAGTCTTCTGGGTGGCAGT | CCCTGGCCAAGGTCATCCATG |

## Real-time RT PCR

## RNA isolation

RNA was precipitated in ethanol from the RNA containing Trizol fraction according to the manufacturer’s instructions. The resulting RNA was DNaseI treated with 5U ml^-1^ of RQ1 DNaseI (Promega) before being extracted by phenol/chloroform and re-precipitated in ethanol.

### Reverse transcription

Total RNAs (1-2µg) were reverse-transcribed using the Superscript III reverse transcription kit (Invitrogen) and random hexamers provided in the kit. The cDNA was diluted and an aliquot of each sample was pooled to give a cDNA standard. This was subsequently serially diluted and used to produce a standard curve. The remaining experimental samples were further diluted so that they could be quantified from the standard curve.

### Real-time PCR

Taqman real-time PCR was performed using 5’Fluorescein and 3’Blackhole quencher labelled probes (Biopolymer Synthesis and Analysis Unit, University of Nottingham), in 25µl reaction volumes using SensiMixdT (Quantace). Triplicate reactions for each experimental sample were performed using an ABI Prism 7000 real-time PCR machine.

Quantification of samples was performed using the standard curve method, with each target gene expression normalised to *gapdh* expression. The mean and standard deviation of 3 independent experiments was plotted and analysed using Graphpad Prism v5/6.

**Co-immunoprecipitations**

For immunoprecipitation of endogenous MED14, Hela cells were cultured in 10cm dishes to ~90% confluency and starved over night in serum-free medium. Cells were harvested directly, or after TPA stimulation for 15 min, and washed twice with ice-cold PBS, then lysed in a modified RIPA buffer (40 mM HEPES PH 7.9, 1% NP-40, 150 mM KCl, 5 mM MgCl_2_, 5 mM NaF, 0.5 mM EDTA, 0.5 mM PMSF, 0.5 mM Na_3_VO_4_, 0.5 mM DTT, 1 µg/ml of leupeptin and pepstatin, 1x phos-stop cocktail, 10% glycerol) at 4°C for 1-2 hours.

Cell extracts were cleared by centrifugation for 15 min at 14K rpm, 4°C and incubated with anti-MED14 antibody (1µg per ml lysate) over night at 4°C on a rotating wheel. Protein G sepharose beads pre-blocked in TNE plus 2% BSA were added and incubated for another 3 hours at 4°C to bind Ag-Ab complexes. Immunoprecipitates were washed four times in lysis buffer and eluted with SDS-PAGE sample buffer. Eluates were heated at 95 °C for 5 minutes and resolved by SDS-PAGE in 7.5% gels. Proteins were transferred to PVDF membrane and detected by immunoblotting.

**Supplementary References**

50. Gille, H., Strahl, T. and Shaw, P.E. (1995) Activation of Ternary Complex Factor Elk-1 by Stress-Activated Protein Kinases. *Curr Biol*, **5**, 1191-1200.

51. Gille, H., Kortenjann, M., Thomae, O., Moomaw, C., Slaughter, C., Cobb, M.H. and Shaw, P.E. (1995) ERK phosphorylation potentiates ELK-1-mediated ternary complex formation and transactivation. *EMBO J.*, **14**, 951-962.

52. Evans, E.L., Saxton, J., Shelton, S.J., Begitt, A., Holliday, N.D., Hipskind, R.A. and Shaw, P.E. (2011) Dimer formation and conformational flexibility ensure cytoplasmic stability and nuclear accumulation of Elk-1. . *Nucl. Acids Res.*, **39**, 6390-6402.

53. Kortenjann, M., Thomae, O. and Shaw, P.E. (1994) Inhibition of v-raf-Dependent c-fos Expression and Transformation by a Kinase-Defective Mutant of the Mitogen-Activated Protein Kinase Erk2. *Mol. Cell. Biol.*, **14**, 4815-4824.

54. Strahl, T., Gille, H. and Shaw, P.E. (1996) Selective response of ternary complex factor Sap1a to different mitogen-activated protein kinase subgroups. *Proc. Natl. Acad. Sci. (USA)*, **93**, 11563-11568.

**Legends to Supplementary Figures**

**Supplementary Figure 1**

**a**) Epitope-tagged Mediator subunits were expressed in HEK293T cells, immunoprecipitated from cell lysates with FLAG or HA antibodies (arrowheads, upper panels) and subjected to *in* *vitro* kinase assays with ^32^P-g-ATP in the absence (-) or presence (+) of recombinant ERK2 (rERK2). Reactions were separated by SDS-PAGE and visualised by phosphor-imaging (lower panels). Arrowheads indicate phosphorylated substrates of expected size; open arrowhead indicates unknown ERK target in MED12 IP.

**b**) Lysates were prepared from HEK293T cells transfected with a vector for FLAG-MED12. Mock (lane 1) or CDK8 co-immunoprecipitates (lane 2) were collected and samples were analysed by SDS-PAGE and immunoblotting for the presence of MED12 (upper), MED14 (middle) and CDK8 (lower panel).

**Supplementary Figure 2**

**a**) Recombinant active ERK (rERK2*) was incubated alone (lane 1), with GST-MED14 SPR (lane 3) or GST (lane 4) captured on glutathione-agarose beads and subjected to an *in* *vitro* ERK kinase assay with ^32^P-γ-ATP. Reactions were separated by SDS-PAGE and visualised by phosphor-imaging (upper panel) and coomassie blue staining (lower panel).

**b**) Full-length HA-tagged MED14 and the deletion mutant MED14D were immunoprecipitated from HEK293 cell lysates and subjected to an *in* *vitro* kinase assay with rERK2* and ^32^P-g-ATP. Reactions were separated by SDS-PAGE and visualised by phosphor-imaging (upper panel, 80% of IP). Relative recoveries were assessed by immunoblotting for HA (lower panel, 20% of IP). Values indicate phosphate incorporation relative to wild-type (*WT*) protein.

**c**) Lysates were prepared from serum-starved (lanes 1 and 3) or TPA-stimulated (+) HeLa cells (lanes 2 and 4). Control (lanes 1 and 2) or CDK8 (lanes 3 and 4) co-immunoprecipitates were collected and analysed in parallel by SDS-PAGE and immunoblotting for the presence of MED14 (upper), MED23 (middle) and CDK8 (lower panel).

**d**) HEK293 cells were transfected with vectors for tagged versions of MED14 and CDK8 as indicated. Nuclear extracts were prepared and CDK8 co-immunoprecipitates were collected with an anti-FLAG antibody and analysed by SDS-PAGE and immunoblotting for the presence of MED14 (upper) and CDK8 (lower panel). Immunoblots of corresponding extracts are shown in the panels below.

**e**) HEK293 cells were transfected with vectors for tagged versions of CDK8, MED12 or MED14 and the proteins were collected from lysates by immunoprecipitation with tag-specific antibodies. Kinase activity towards the MED14-SPR domain in the immunoprecipitates was assayed *in* *vitro* either alone (lanes 4) with the CDK inhibitor Roscovitine (lane 5) or in conjunction with rERK2* (lanes 6-8). ERK phosphorylation of GST-MED14-SPR is inhibited by a combination of ERK inhibitors (compare lanes 2 and 3).

**Supplementary Figure 3**

HEK293 cells transfected with expression vectors for HA-MED14 alone (1-2) or with active versions of MEKK (3-4), PI3K (5-6), Akt/PKB (7-8), RhoA (9-10), Rac1 (11-12), cdc42 (13-14), SEK (15-16) or PAK1 (17-18) were serum starved for 16h and treated with the MEK inhibitor U0126 or DMSO control for 2h. Nuclear extracts were prepared and MED14 expression, S986 phosphorylation and ERK activation were monitored by immunoblotting. Activator expression was subsequently confirmed where possible by re-probing with the antibodies indicated (lower set of panels).

**Supplementary figure 4**

Quantitative RT-PCR analysis of MED14 knockdown on *mcl1* RNA expression in HeLa cells following serum stimulation. Data represent mean values from 3 experiments each performed in triplicate. Multiple 2-tailed t-tests to compare means (control v MED14 KD) at each time point showed no significant differences.

**Supplementary Figure 5**

(**a**) NIH3T3 cells were transfected with a Gal4-Luc reporter, Gal4(1-143) or Gal-Elk fusion protein, expression vectors for MED14, MED14-S986A or the corresponding vector (pRK5) and either TK-renilla (upper) or UbC-Renilla (lower) control plasmid. After serum-starvation for 24h cells were left untreated (-) or stimulated with TPA for 6h (+). Data are mean Renilla values from two representative experiments, each with triplicate points.

(**b**) HEK293 cell lysates used in one representative experiment from the reporter assays in figure 5a were separated by SDS-PAGE and MED14 expression was determined by immunoblotting (LH panels). To compare MED14 expression levels in NIH3T3 cells, ten-fold more of each vector was transfected alone in order to detect MED14 expression by SDS-PAGE and immunoblotting (RH panels). The loading controls were actin and tubulin respectively.

**Supplementary Figure 6**

HCT116 cells expressing MED14 shRNA or control cells were serum starved or starved and treated with serum for 30 min. RNAPII occupation and CDK8 recruitment (upper graphs), MED14 recruitment and S986 phosphorylation (middle), pS5-CTD and pS2-CTD modifications (bottom) at the c-*fos* gene were determined by ChIP assay. Data represent mean values from 3 experiments.

**Supplementary Figure 7**

(**a**) HEK293 cells were transfected with vectors for tagged versions of MED14, Elk-1 and Elk-dbl, which lacks aas 308-21, as indicated. Cells were serum-starved (lanes 1, 2, 5), treated with EGF alone (lanes 3 and 6) or with EGF after prior addition of the p38^mapk^ inhibitor SB290190 (lanes 4 and 7). Lysates were prepared, MED14 co-immunoprecipitates were collected with an anti-HA antibody and analysed by SDS-PAGE and immunoblotting for the presence of Elk-1 (upper) and MED14 (lower panel). Immunoblots of corresponding lysates are shown in the panels below.

(**b**) MED23 *wt* and -/- MEFs were serum-starved for 16h or starved and treated with TPA for 15 min. Cell lysates were prepared and 5% of each was analysed for ERK activation and MED14 phosphorylation by SDS-PAGE and immunoblotting with the antibodies indicated. Due to low sensitivity of the MED14 antibody, relative MED14 levels were assessed in MED14 immunoprecipitates from 90% of the lysates (second panel).


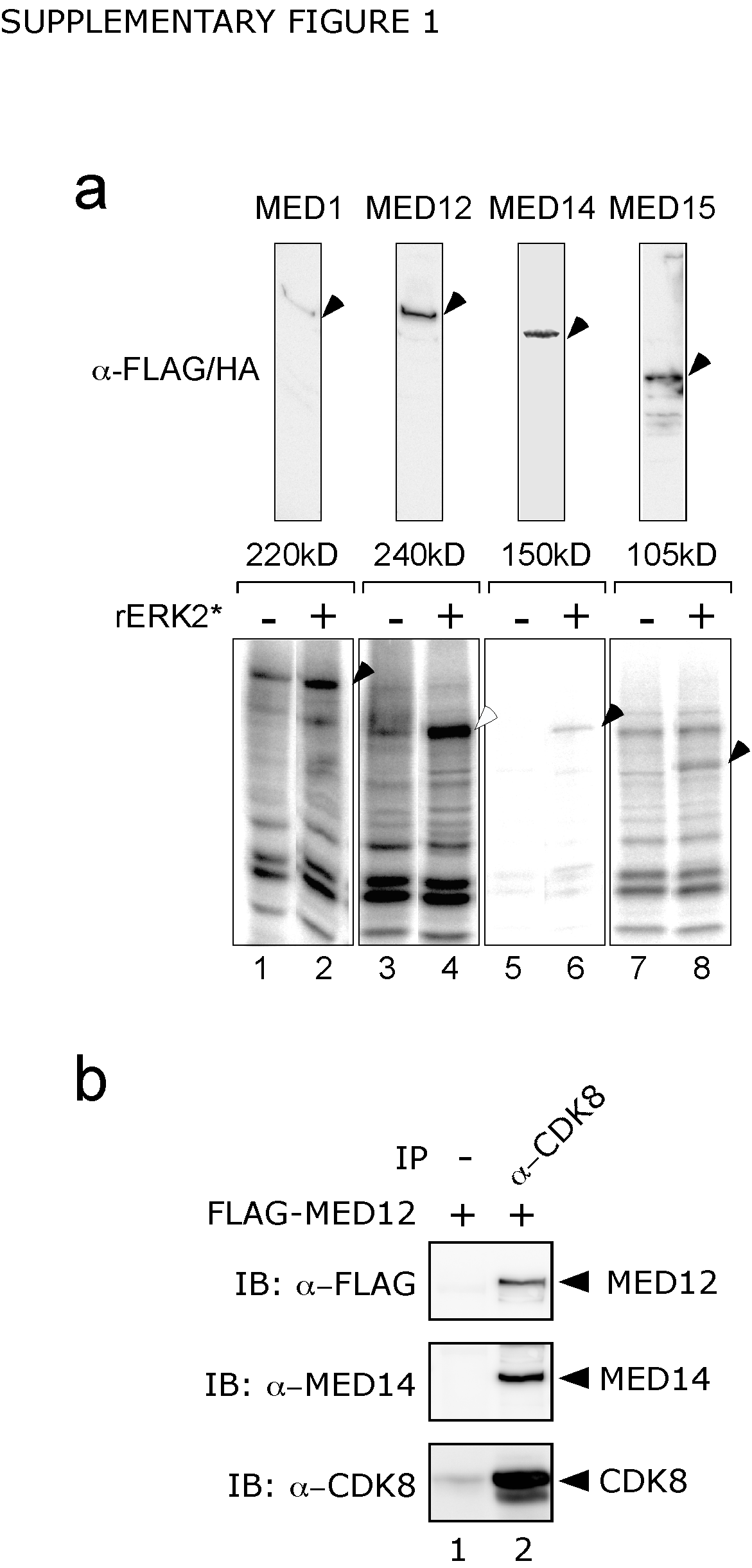


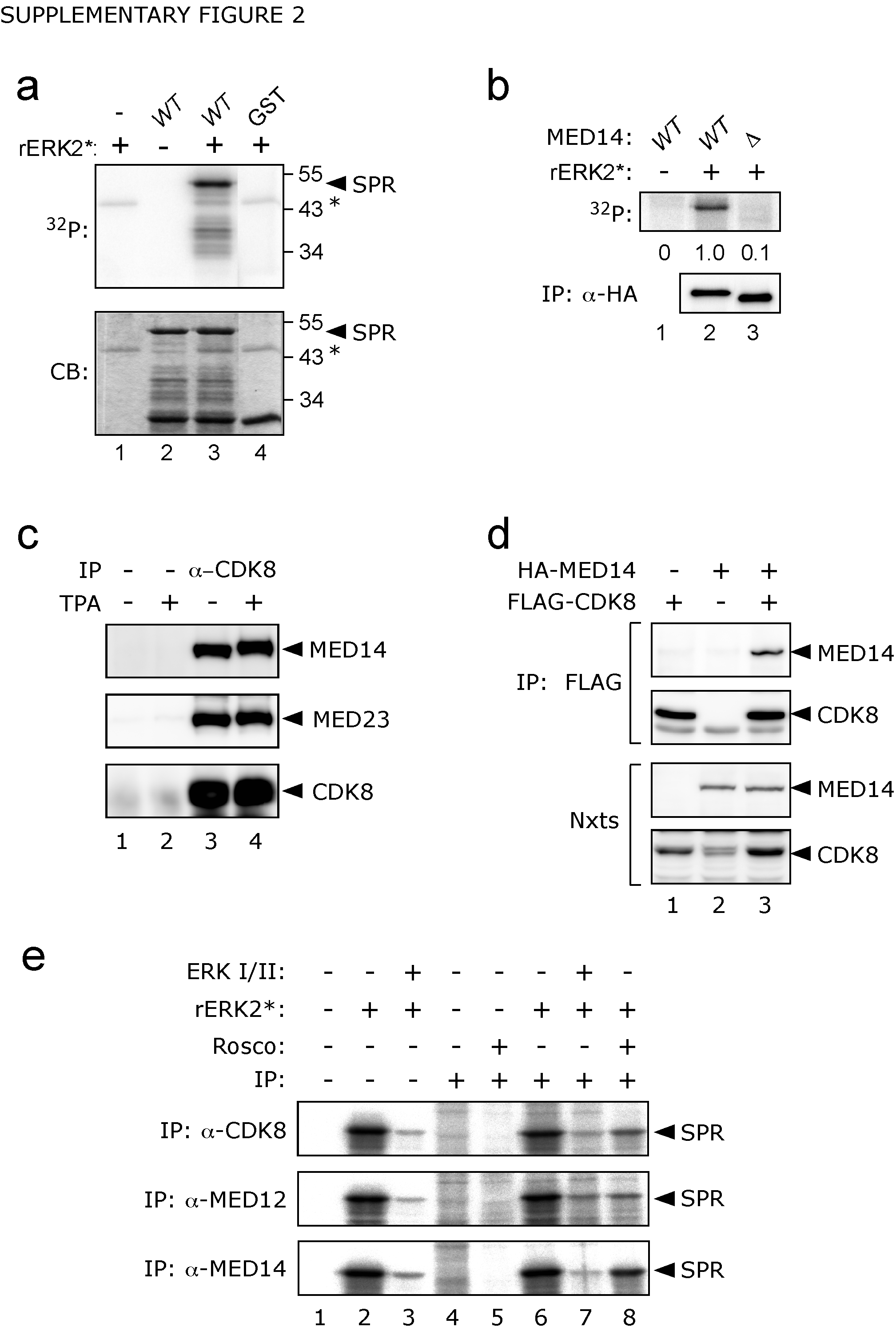

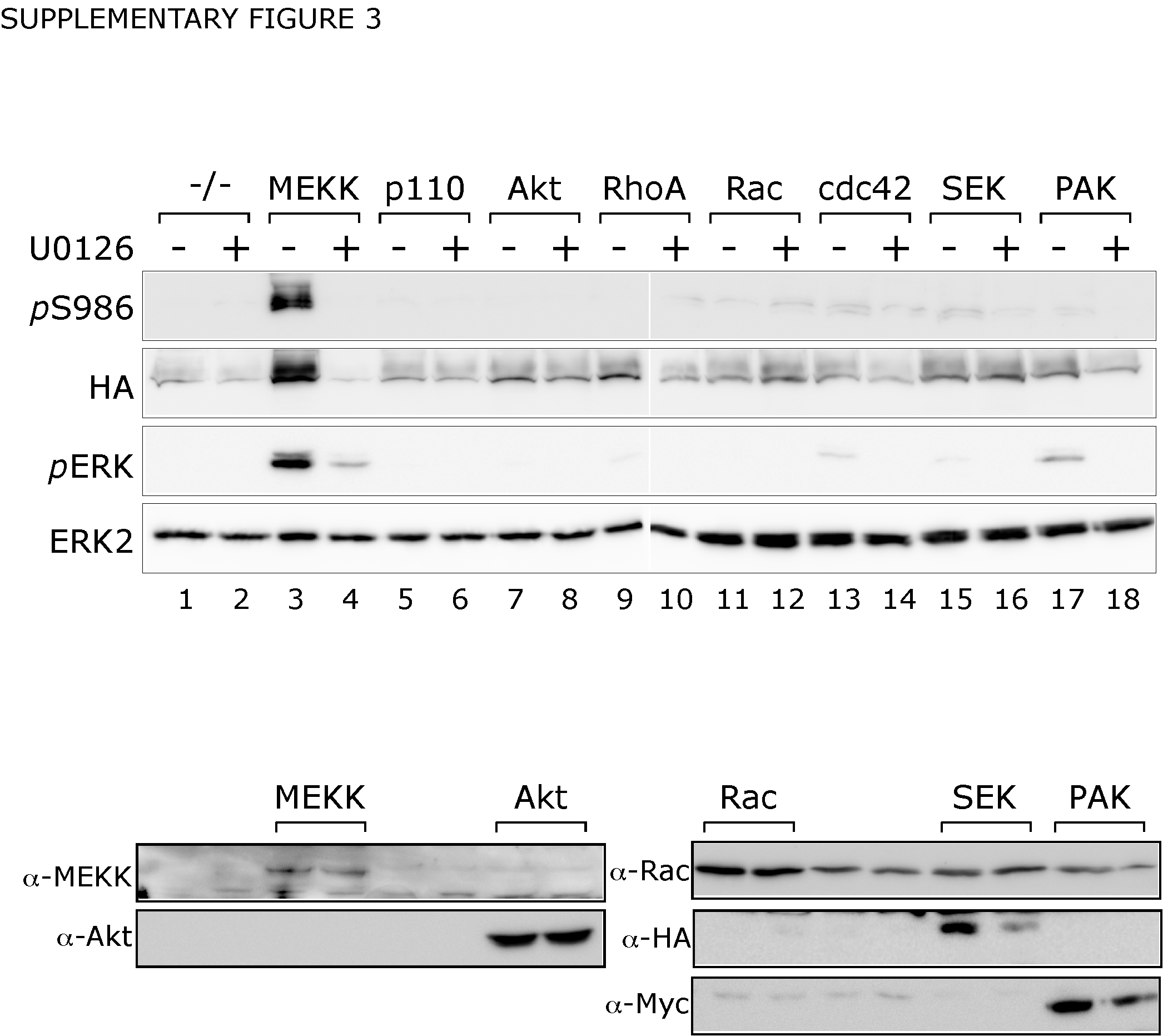


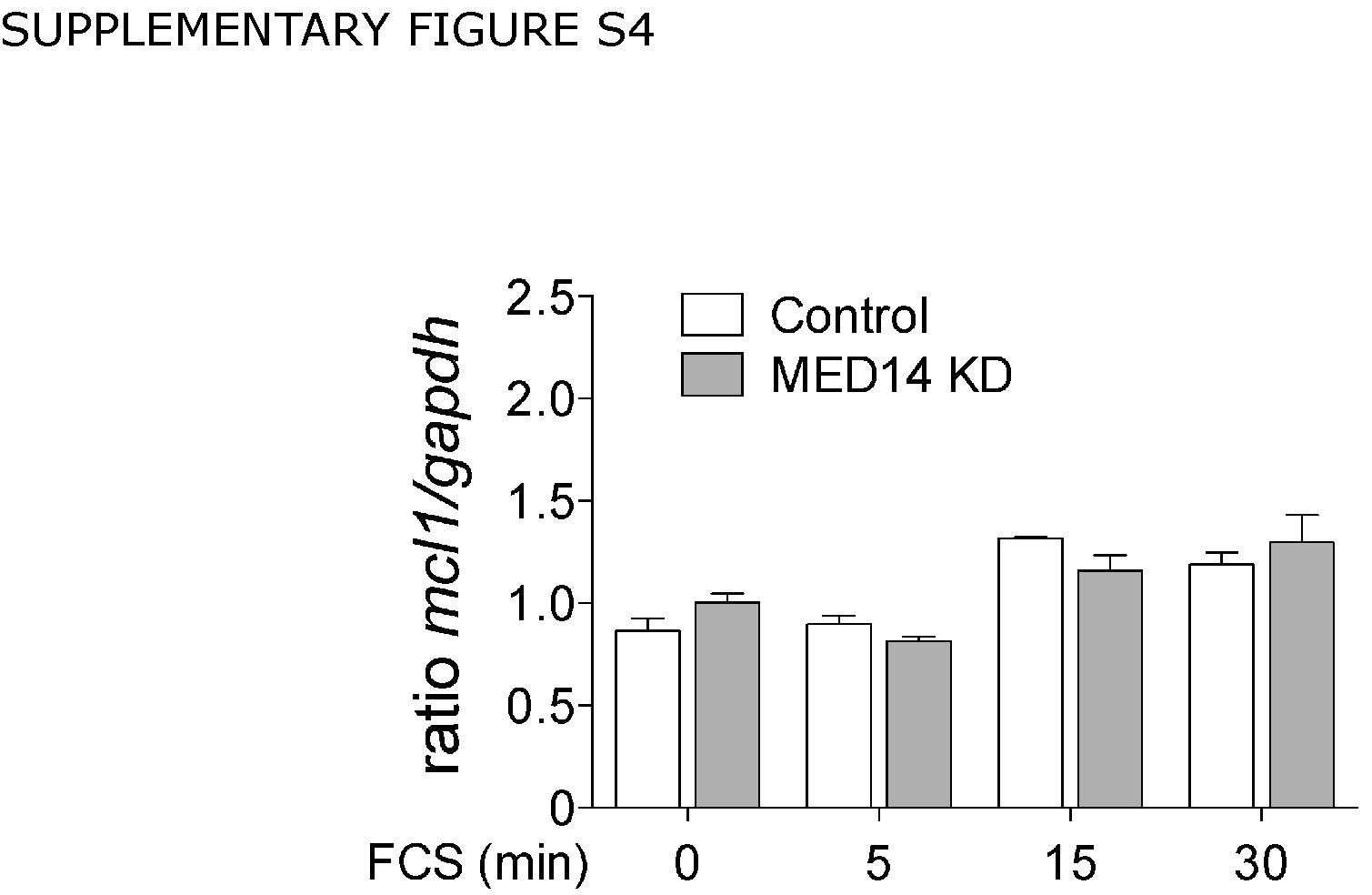

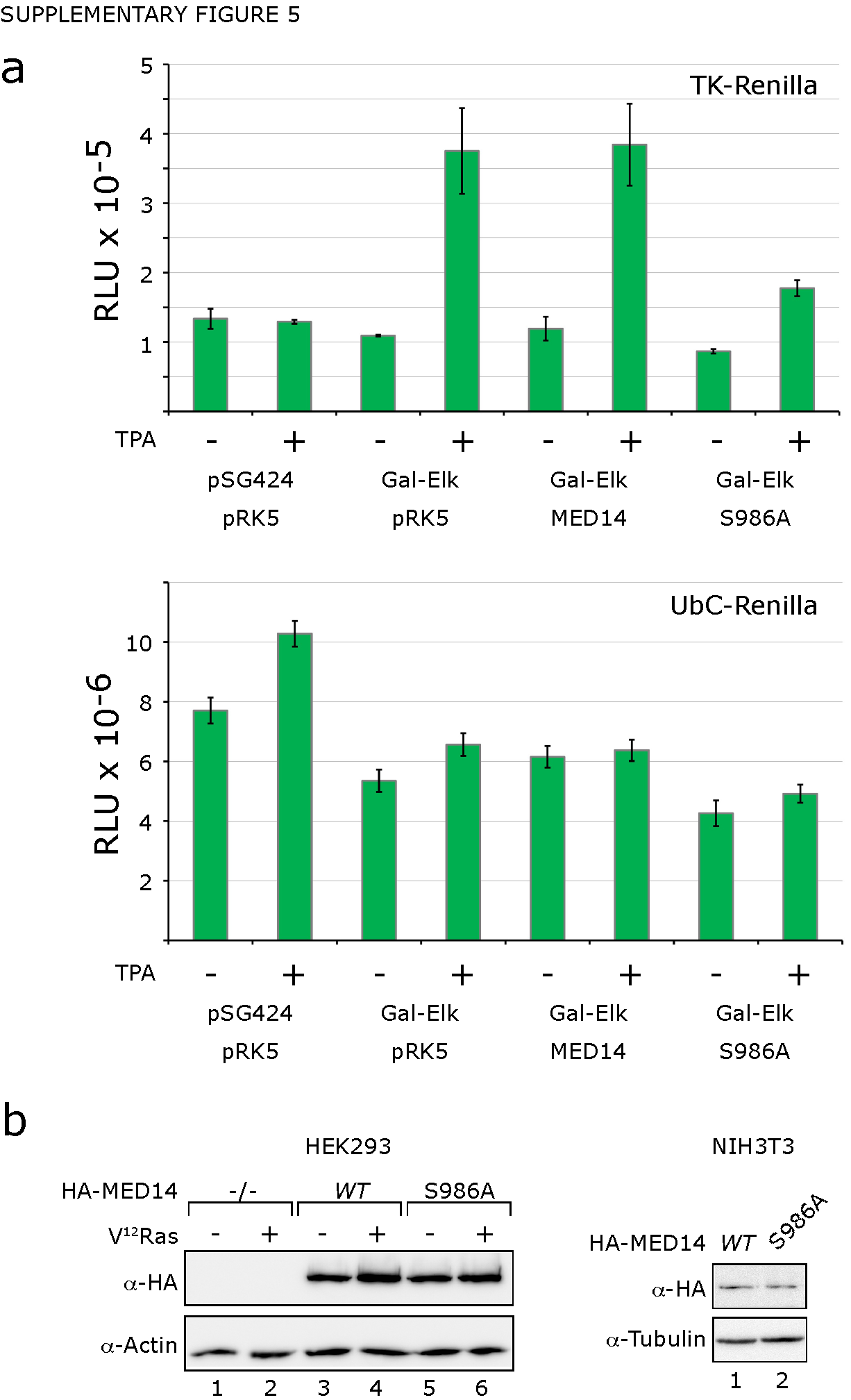

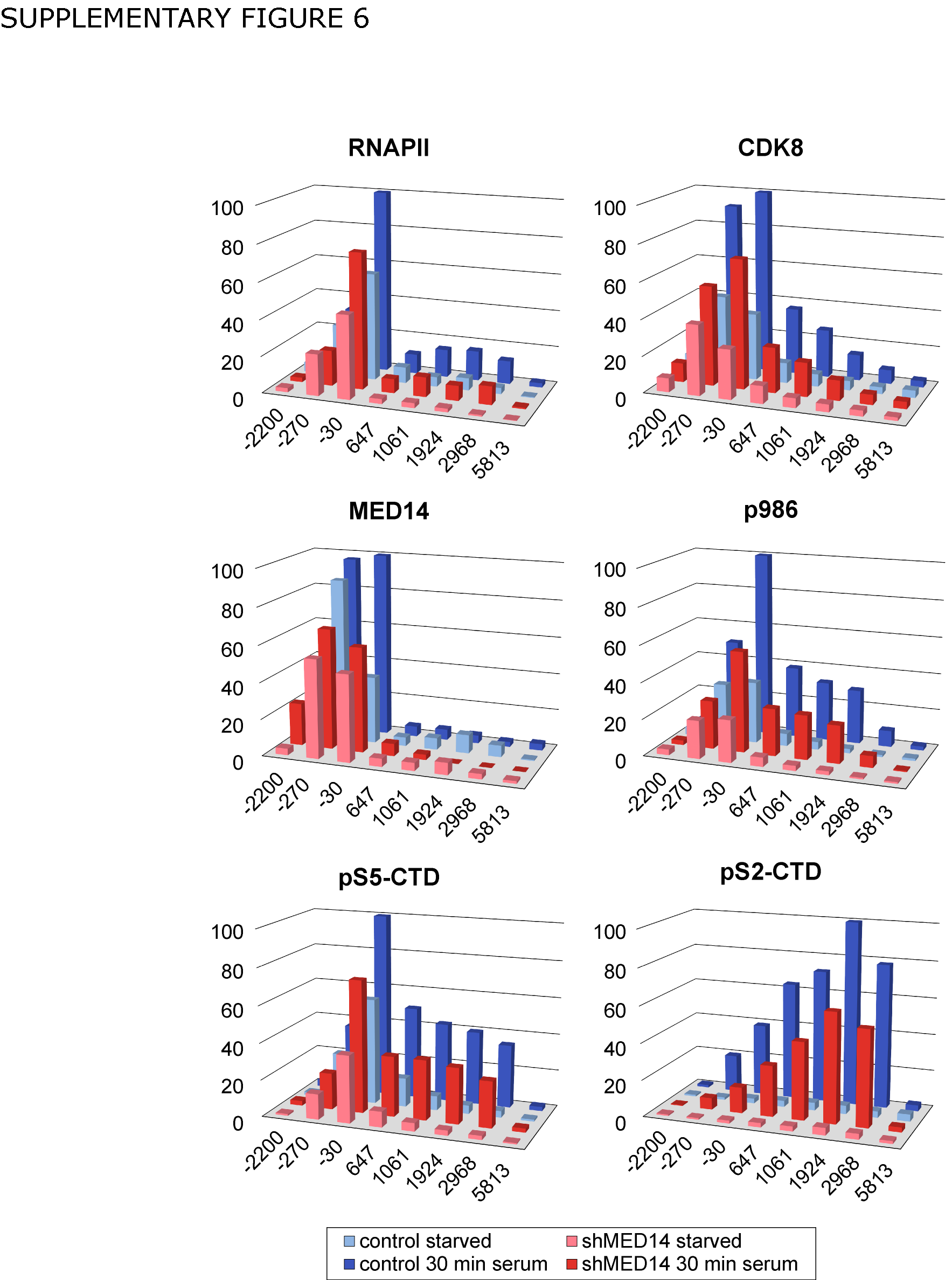

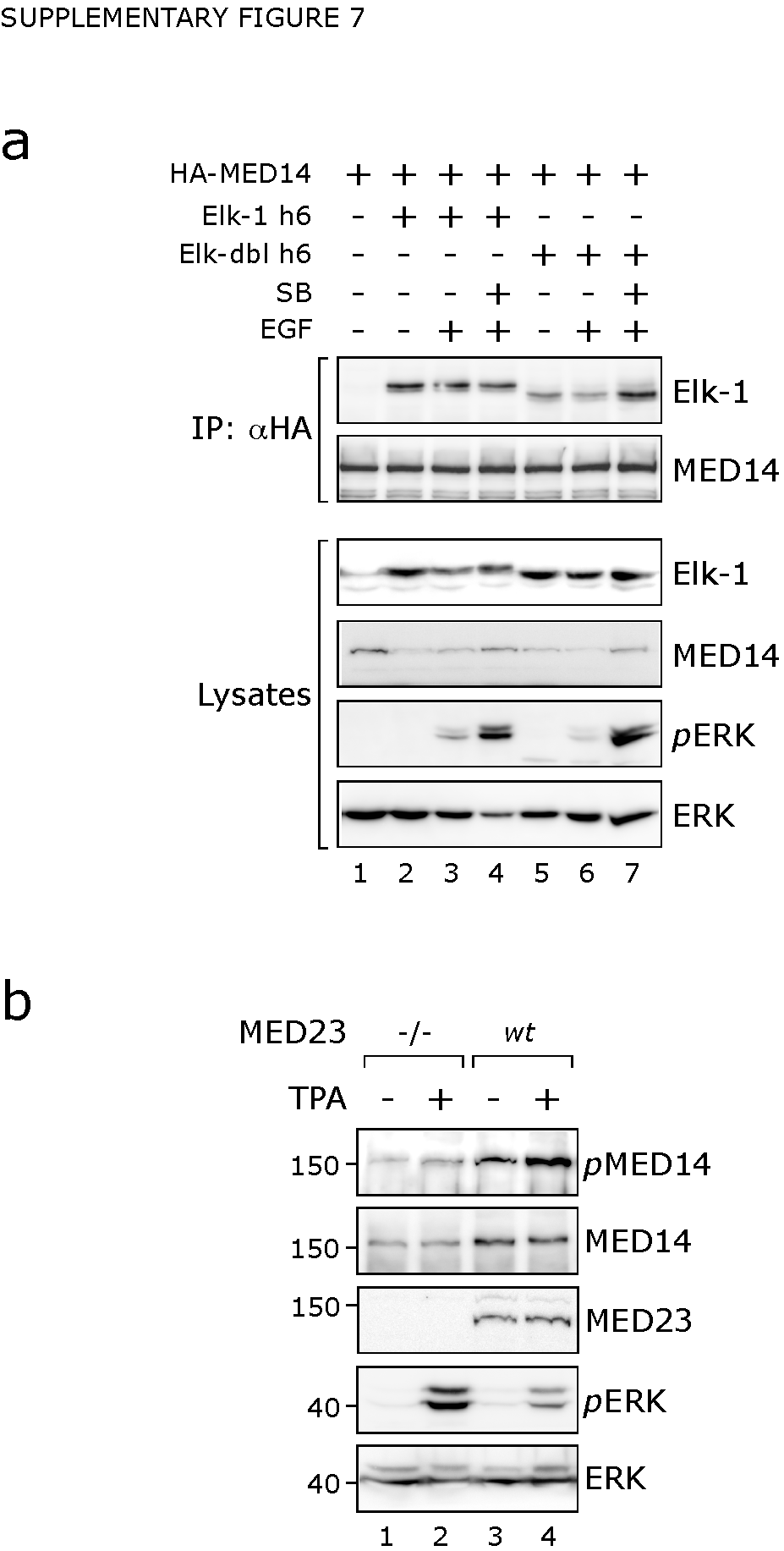

Supplement: Supplementary Data [file supp_gkt837_nar-00847-x-2013-File012.doc]
